# Frequent gene conversion events between the X and Y homologous chromosomal regions in primates

**DOI:** 10.1186/1471-2148-10-225

**Published:** 2010-07-23

**Authors:** Mineyo Iwase, Yoko Satta, Hirohisa Hirai, Yuriko Hirai, Naoyuki Takahata

**Affiliations:** 1 The Center for the Promotion of Integrated Sciences, The Graduate University for Advanced Studies (Sokendai), Shonan village, Hayama, Kanagawa 240-0193, Japan; 2 Department of Evolutionary Studies of Biosystems, The Graduate University for Advanced Studies (Sokendai), Shonan village, Hayama, Kanagawa 240-0193, Japan; 3 Primate Research Institute, Kyoto University, Inuyama, Aichi 484-8506, Japan; 4 The Graduate University for Advanced Studies (Sokendai), Shonan village, Hayama, Kanagawa 240-0193, Japan

## Abstract

**Background:**

Mammalian sex-chromosomes originated from a pair of autosomes. A step-wise cessation of recombination is necessary for the proper maintenance of sex-determination and, consequently, generates a four strata structure on the X chromosome. Each stratum shows a specific per-site nucleotide sequence difference (*p-*distance) between the X and Y chromosomes, depending on the time of recombination arrest. Stratum 4 covers the distal half of the human X chromosome short arm and the *p*-distance of the stratum is ~10%, on average. However, a 100-kb region, which includes *KALX *and *VCX*, in the middle of stratum 4 shows a significantly lower *p*-distance (1-5%), suggesting frequent sequence exchanges or gene conversions between the X and Y chromosomes in humans. To examine the evolutionary mechanism for this low *p*-distance region, sequences of a corresponding region including *KALX*/*Y *from seven species of non-human primates were analyzed.

**Results:**

Phylogenetic analysis of this low *p*-distance region in humans and non-human primate species revealed that gene conversion like events have taken place at least ten times after the divergence of New World monkeys and Catarrhini (*i.e*., Old World monkeys and hominoids). A *KALY*-converted *KALX *allele in white-handed gibbons also suggests a possible recent gene conversion between the X and Y chromosomes. In these primate sequences, the proximal boundary of this low *p*-distance region is located in a *LINE *element shared between the X and Y chromosomes, suggesting the involvement of this element in frequent gene conversions. Together with a palindrome on the Y chromosome, a segmental palindrome structure on the X chromosome at the distal boundary near *VCX*, in humans and chimpanzees, may mediate frequent sequence exchanges between X and Y chromosomes.

**Conclusion:**

Gene conversion events between the X and Y homologous regions have been suggested, mainly in humans. Here, we found frequent gene conversions in the evolutionary course of primates. An insertion of a *LINE *element at the proximal end of the region may be a cause for these frequent conversions. This gene conversion in humans may also be one of the genetic causes of Kallmann syndrome.

## Background

The mammalian sex chromosomes (X and Y chromosomes) differentiated from a pair of autosomes (proto-sex chromosomes) more than 200 million years (myr) ago, before the divergence of monotremes from other mammals [1,2]. The physiological and biological differentiation of proto-sex chromosomes began with the emergence of two or more sex-determining genes, including *SRY *(sex-determining region on Y) and *RBMY *(RNA binding motif protein, Y-linked), on one of the proto-sex chromosomes, the proto-Y chromosome. The tight linkage of sex-determining genes on the proto-Y chromosome is required for normal development of male characters or male determination [3], and the suppression of homologous recombination produced the tight linkage of male-sex-determining genes on the proto-Y chromosome. Through suppression of homologous recombination, nucleotide substitutions accumulated on each chromosome, leading to the emergence of X- and Y-specific regions on each sex chromosome. Eventually, the increased divergence between the X and Y chromosomes led to a complete cessation of recombination between them, leading to the rapid degradation of the Y chromosome. This degradation arises primarily from the loss of a mechanism that avoids the accumulation of deleterious mutations on the Y chromosome [4,5].

Lahn and Page [6] first proposed a scenario to explain the process of sex chromosome differentiation. The extent of nucleotide divergence per synonymous site (*K*_S_) for 19 X-Y pairs of genes in humans or squirrel monkeys varied greatly from 0.05 at *ARSE*/*ARSEP *to 1.25 at *SRY*/*SOX3*. In addition, the *K*_S _of the 19 pairs decreased with position on the X chromosome from the long arm tip to the short arm tip. Based on this variation, the 19 pairs were classified into four distinct categories, or strata. Stratum 1 contains three genes with *K*_S _> 0.9, stratum 2 contains two genes with *K*_S _of approximately 0.5, and strata 3 and 4 comprise seven genes with *K*_S _= ~0.2, and *K*_S _= ~0.1, respectively. The *K*_S _values in strata 1 and 2 suggest that recombination between X and Y chromosomes ceased before the divergence of monotremes from other mammals for stratum 1, and before the divergence of marsupials and eutherian mammals for stratum 2. The observed *K*_S _values of ~0.2 and ~0.1 also suggest that stratum 3 formed before the mammalian divergence and that stratum 4 formed in the primate lineage after the divergence of prosimian and simian primates. To confirm whether this step-wise divergence between X and Y chromosomes is observed in other non-coding regions on the X and Y chromosomes, Iwase et al. [7] compared nucleotide sequences in the distal half of the short arm of the human X chromosome with a homologous region on the Y chromosome. This analysis revealed a step-wise change in the *p*-distances and confirmed the scenario of sex chromosome differentiation proposed by Lahn and Page [6]. However, some *p*-distances within stratum 4, where the *p*-distance is ~0.1 on average, were significantly lower (0.01~0.05) (Additional file 1) [7]. This low *p*-distance region has been reported independently in other studies [8]. The region is ~100 kb long and encompasses the *KALX *to *VCX2 *genes in humans (Additional file 1). The cause for this low *p*-distance has been argued to be gene conversion [8].

*KALX*, which is one of the genes responsible for Kallmann syndrome [9-12], is located on the X chromosome, and is composed of 14 exons, encompassing a 210-kb region. The gene encodes an extracellular matrix glycoprotein of ~100 kDa which is called anosmin-1 and is expressed in the kidneys and in multiple embryonic tissues [9,13,14]. Anosmin-1 is conserved in a wide range of animals, from nematodes to humans [14-17] suggesting the functional importance of the protein. A deletion in or of the *KALX *gene results in an X-linked recessive form of Kallmann syndrome, whose typical symptoms are anosmia, small genitalia, and sterility. The defective gene product prevents the migration of gonadotropin-releasing hormone (GnRH) neurons and olfactory neurons to the hypothalamus [17-21]. A homolog of *KALX*, *KALY*, is located on the long arm of the Y chromosome, but because of deletions and nonsense mutations the gene is a pseudogene [22,23]. Since *KALX *and *KALY *are in the middle of stratum 4, the two genes began to differentiate after simian primates diverged from prosimians [6].

*VCX *and *VCY *form a relatively small gene family, with four members of *VCX *and two of *VCY *being identified in humans. Each member of the *VCX *gene family has three or four exons and is 1320 ~1880 bp in length, whereas *VCY *genes have two exons and are 742 bp long. All members of *VCX *and *VCY *are expressed exclusively in male germ cells [24]. *VCX *and *VCY *sequences show a high degree of sequence similarity within each gene family as well as between genes on the X and Y chromosomes [25]. Sequence identity within *VCY *is due to frequent gene conversion between palindrome arms [26,27].

The purpose of this study was to examine the evolutionary mechanism that produces the low *p*-distance region within stratum 4 in non-human primates. To this end, we compared nucleotide sequences of corresponding regions between X and Y chromosomes of several primates. The analysis showed that relatively frequent gene conversions between the X and Y chromosomes have occurred independently in each of the primate lineages. This frequent gene conversion is argued to be a result of genomic structure, such as repetitive elements at the boundary of the region.

## Results

### Comparison of genome sequences reveals low *p*-values in stratum 4

**Figure 1:**
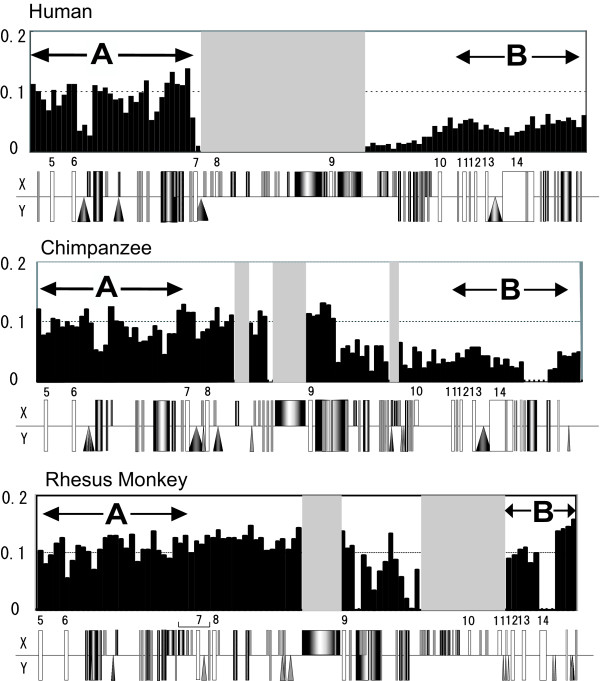
**Gene arrangement and comparison of the *p*-distances between primate *KALX *and *KALY***. The centromeric boundaries of the low *p*-distance region in humans (top), chimpanzees (middle), and rhesus monkeys (bottom) are shown. When a part of the *KALY *sequence was deleted, the region is shown in gray. These *p*-distances (y-axis) were computed in non-overlapping windows of 500-bp each. Repetitive elements detected using Repeat masker and NCBI Map Viewer are indicated (square, triangle, and circle) under each *p*-distance graph. Small numbers on the diagram indicate the position of exons (white rectangles). A bracket in the rhesus monkey diagram indicates an inversion. Regions A and B used for phylogenetic analysis are marked by two-headed arrows.

Window analysis of *p*-distances between X and Y chromosomes in chimpanzees shows a low *p*-distance region on the X chromosome that corresponds to a similar low *p*-distance region on the X-chromosome in humans. With the exception of the centromeric region containing *KALX *or *KALY *(Additional file 1), the corresponding regions show similar *p*-distances in the two species. The sub-region values are ~10 kb with *p *= 0.05, ~5 kb with *p *= 0.10, and ~70 kb with *p *= 0.05, and the boundaries of the chimpanzee sub-regions are almost identical to those in humans (Additional file 1). A part of the *KAL *coding region, however, differs between the two species. In chimpanzees, a region covering the middle of intron 9 to the 3' flanking region of *KALX *shows a divergence with *KALY *of *p *= 0.05. An adjacent region shows an identical nucleotide sequence (*p *= 0), whereas the corresponding regions in humans have values of *p *= 0.01 and 0.05, respectively (Figure 1B, Additional file 1). It should be noted that the boundaries of the above two sub-regions differ between the two species (Figure 1B). In addition, the organization of the *KALY *gene (Figure 1A) also differs between the two species: human *KALY *has a large deletion containing introns 7 to 9, whereas chimpanzee *KALY *retains all exons and introns, with the exception of exon 3, which is also deleted in human *KALY*.

The entire rhesus monkey *KALY *is not available from data obtained in the present study or sequence databases, but the available *KALY *sequence shows a deletion from intron 9 to 11 (Figure 1A). In the 50 kb sequence obtained in the present study the *p*-distance between *KALY *and *KALX *is ~0.10, while in a 1.5 kb region including exon 14 and the 3' flanking region the value is ~0.01 (Figure 1B). We also found an inversion on the X chromosome from intron 6 to exon 8 with a length of ~3.5 kb in rhesus monkeys. The low *p*-distance region in rhesus monkeys, chimpanzees, and humans shows some species-specific patterns of *p *values, suggesting that the timing of gene conversion between X and Y chromosomes is species-specific in each of the lineages leading to the three species.

### Gene conversion in humans, chimpanzees, and rhesus monkeys

**Figure 2:**
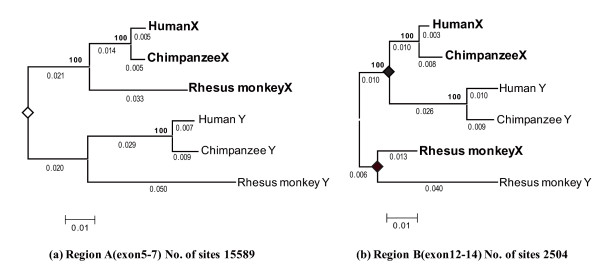
**The phylogeny of Regions A and B of *KALX *and *KALY *in humans, chimpanzees, and rhesus monkeys**. The phylogenetic trees for both Region A and Region B are based on *p*-distances. The numbers of sites used for constructing the tree are indicated at the bottom right of each tree. An open diamond at a node indicates *KALX *and *KALY *differentiation, and the number at the node is the bootstrap value in 500 replications. Filled diamonds indicate gene conversion events. The branch lengths are shown below each branch. A scale bar for the *p*-distance is indicated at the bottom left of the tree. Sequences on the X and Y chromosome are represented by bold and regular fonts, respectively.

The formation of a low *p*-distance region in the middle of stratum 4 is likely due to gene conversion between the X and Y chromosomes. Therefore, we examined how often and where in the *KAL *genes these conversions occurred. First, to determine the position of the most recent conversion between *KALX *and *KALY *in humans, chimpanzees, and rhesus monkeys, we examined the boundary of the low *p*-distance region by phylogenetic analysis. We constructed 50 phylogenetic trees for 2 kb-fragment windows with a sliding overlap of 1 kb. Based on the topologies of these trees, the *KAL *coding region was classified into two sub-regions: one encompassing exons 5 to 7 (Region A in Figure 1, Figure 2A) and the other encompassing exons 12 to 14 (Region B in Figure 1, Figure 2B). The region from intron 7 to 11 was not included in the analysis because of independent deletions in the *KALY *gene of humans and rhesus monkeys. The phylogenetic trees of Region A show the history of stratum 4. Recombination between X and Y chromosomes ceased before the divergence of Old World monkeys and hominoids (apes and humans) and no further exchanges occurred. Therefore, sequences from the three primates show the reciprocal monophyly of *KALX *and *KALY*. In contrast, Region B shows rather species-specific clustering, suggesting independent gene conversion in these species.

Second, to determine the timing of the most recent conversion in these lineages, the per-site nucleotide divergence (*d*) on the branches leading to the X and Y chromosome sequences in Regions A and B were calculated. Since the nucleotide substitution rates of the X and Y chromosomal genes differ [28], *d *values on the X and Y chromosomes are estimated separately on the tree (Figure 2A and 2B). The average *d *value on the lineage leading to *KALX *(*d*_X_) and *KALY *(*d*_Y_) in rhesus monkeys and hominoids from a common ancestor of these species is given by *d*_X _= 0.023 ± 0.007 and *d*_Y _= 0.041 ± 0.006 for Region A, and *d*_X _= 0.023 ± 0.003 and *d*_Y _= 0.046 ± 0.001 for Region B. The values of *d*_X _and *d*_Y _in Region A are similar to those in Region B. This is consistent with the gene conversion that does not affect species divergence. The divergence re-estimated using both sequences in Regions A and B gives *d*_X _= 0.023 ± 0.005 and *d*_Y _= 0.044 ± 0.005. Assuming a divergence time between rhesus monkeys and hominoids of 30 myr [29], the average substitution rate of *KALX *and *KALY *is 0.78 ± 0.18 × 10^-9 ^and 1.45 ± 0.17 × 10^-9 ^per site per year, respectively. Based on these substitution rates, the timing of gene conversion in Region B for rhesus monkey X and Y chromosomes was estimated to be 17~28 myr ago and that for hominoids was 19~24 myr ago (Figure 2A and 2B). Gene conversion between the X and Y chromosomes could also be observed in other hominoid species, such as gorillas and gibbons.

### Gene conversion in other primate species

To test whether gene conversion can be observed in other primates, specifically gorillas, gibbons, New World monkeys, and ring-tailed lemurs, the chromosomal positions of *KALX *and *KALY *in the genomes of these primates were confirmed by FISH with DAPI (4,6-diaminido-2-phenylidole) staining of the chromosomes. A single copy of *KAL *was detected on the X and Y chromosomes of each species, with the exception of lemurs (Additional file 2). The observation is consistent with the origin of stratum 4 in simian primates. We sequenced the low *p*-distance 7 kb region, encompassing exon 10 to exon 14 of the *KALX *and *KALY *genes from gorillas and gibbons. However, *KALY *gene sequences were not available for ring-tailed lemurs or New World monkeys. Based on the obtained sequences, phylogenetic trees were constructed separately for Regions A and B using the ring-tailed lemur *KALX *as the out-group (Additional file 3) in both trees. The phylogenetic tree of Region A shows again a typical topology of stratum 4: recombination between X and Y halted after the divergence of prosimian and simian primates, producing the reciprocal monophyly of *KALX *and *KALY *of simian primates. In Region B, in contrast, the clusters were taxon-specific. This result suggests that multiple gene conversions occurred in Region B, at least three times; once in the common ancestor of chimpanzees, gorillas and humans, once in an ancestor of gibbons, and once in the lineage of rhesus monkeys. In addition, New World monkey *KALX *appears as an out-group of Catarrhini (Old World monkeys and hominoids) *KALX *and *KALY*, reflecting an ancient gene conversion between X and Y in the stem lineage of Catarrhini.

**Figure 3:**
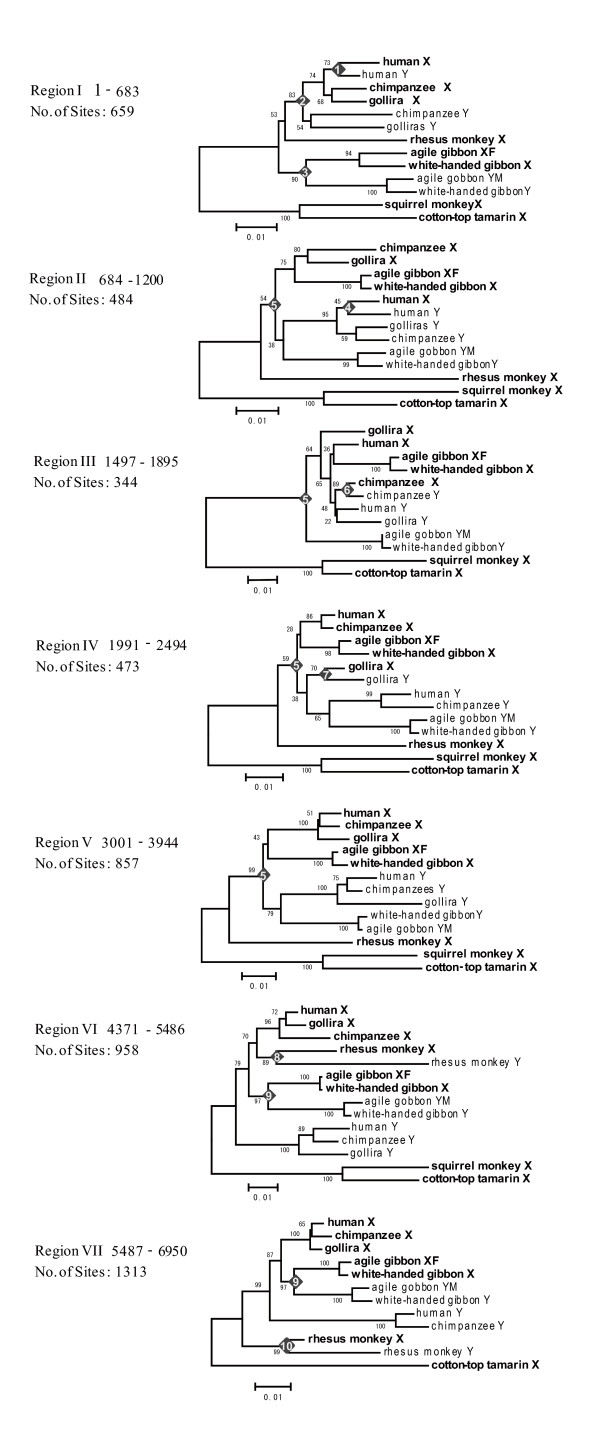
**The phylogenetic tree for primate *KAL *Region B sequences**. Phylogenetic trees were constructed by the NJ method using a window of 500-bp with a 100-bp overlap. In total, 20 trees were obtained. These trees were divided into seven groups by topology. Trees for the seven sub-regions are indicated. Filled diamonds indicate gene conversion events. The number inside the diamond indicates the conversion event given in Figure 6. Sequences on the X and Y chromosome are represented by bold and regular fonts, respectively.

To understand whether or not there are hot spots of conversion in the B region, we analyzed the 7 kb region in detail. We investigated a total of 20 NJ trees with a sliding window width of 500 bp and an overlap of 100 bp for the B region. Based on the topologies of the 20 trees, the B region was further classified into seven sub-regions, each of which shows signatures of gene conversion (Figure 3). Region I shows at least three conversion events: one in gibbons, another in the stem lineage leading to humans, chimpanzees and gorillas, and the third in humans. Similarly, conversions occurred twice in Region II, once in Region III and in Region IV, twice in Region VI, and once in Region VII. In total, at least 10 gene conversions were detected. However, no conversion has occurred in the A region since the divergence of Catarrhini from New World monkeys. These observations strongly imply that the entire B region is prone to gene conversion and that some factors that enhance gene conversion may be present at the boundary between the A and B regions.

### Gene conversion between *KALX *and *KALY *in white-handed gibbons

**Figure 4:**
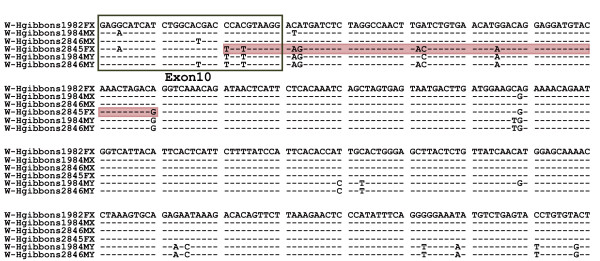
**A recent gene conversion found in the white-handed gibbon *KALX *allele**. Alignment of four *KALX *and two *KALY *alleles in the white-handed gibbon. A dash indicates nucleotides identical to the *KALX *allele sequence shown on top and the black framed rectangle indicates the region of exon 10. One *KALX *allele (2845FX) has a sequence identical to a limited region of approximately 70 to 140 bp in *KALY *(red shaded).

Numerous (at least ten) independent inter-chromosomal gene conversions between *KALX *and *KALY *have occurred during primate evolution. The direction of gene conversion was inferred from the phylogenetic analysis. Among the conversions, five were from *KALY *to *KALX *and the remaining five were in the opposite direction. We also identified an example of ongoing gene conversion from Y to X chromosomes in white-handed gibbons. The alignment of four *KALX *and two *KALY *sequences from four (two females and two males) white-handed gibbons (Figure 4) indicates that the 70 bp sequence starting from the end of exon 10 in one of the *KALX *alleles (white-handed gibbon 2845FX) is identical to a *KALY *sequence (white-handed gibbon 2846 MY). Since these *KALX *and *KALY *sequences differ by eight substitutions, it is unlikely that all eight substitutions are the result of recurrent substitutions on a particular region of the *KALX *allele sequence. This observation is likely an example of a recent gene conversion in this region in a non-human primate.

### Repetitive elements in the region

To infer the molecular mechanism for the frequent gene conversions in the B region, repetitive sequences such as *LINE*, *SINE*, and simple repeats, which are known to be involved in sequence exchanges, were searched for at the boundary of the low *p*-distance region of humans, chimpanzees, and rhesus monkeys. *LINE-1 (L1) *retrotransposons represent at least 17% of the human genome [30], and the number of these elements in the low *p*-distance region does not differ from the average over the entire lengths of the X and Y chromosomes. *KALX *and *KALY*, however, share *L1 *elements (about 3 kb) near the boundary (Figure 1A) of the low *p*-distance region in intron 9, although human *KALY *lacks the *LINE *due to a large deletion encompassing introns 7 to 9.

**Figure 5:**
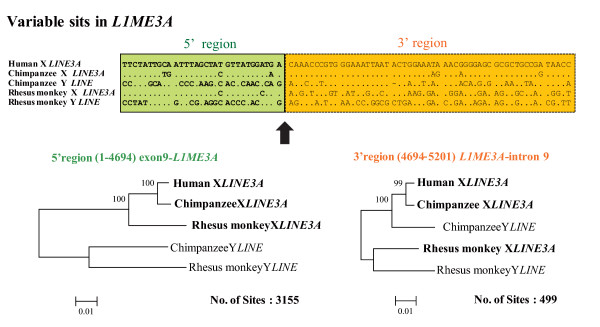
**A *LINE *element is present in the converted region**. (a) *L1 *(*LINE3A) *sequences in intron 9 of *KALX *and *KALY. *Two regions which give different phylogenies are indicated by different colors (green and orange). An arrow indicates the boundary of the two regions. (b) A NJ tree based on *p*-distance in the 5' and 3' regions of these *L1 *(*LINE3A*) sequences. Numbers at the nodes indicate bootstrap probability. Scale bars and the number of sites used for construction of the tree are indicated at the bottom of each tree.

The *LINE *element shared among primate *KALX *and *KALY *has been annotated as *LINE3A*. This *LINE *element is likely to have been inserted before the formation of stratum 4, and it has been a breakpoint for gene conversion. An alignment and phylogenetic analyses of the *LINE3A *sequences revealed the boundary between Regions A and B was located in the sequence and the phylogeny of *LINE3A *showed a reciprocal monophyly in the 5' half of the X and Y sequences but a species-specific clustering in the 3' half of the X and Y sequences (Figure 5). This data suggests the occurrence of at least two independent gene conversions (one in rhesus monkeys and the other in hominoids) in a similar position in *LINE3A*.

### *VCX *and *VCY*

The low *p*-distance region in stratum 4 ranges from *KALX *to *VCX2 *in human and chimpanzee sequences (Additional file 1). Frequent gene conversions between paralogs on the same chromosome are well known among the *VCX *and *VCY *genes. In particular, gene conversion between *VCY *genes is due to the P8 palindrome in humans and chimpanzees [26]. In contrast to *KAL *sequences, *VCY *sequences are only available for humans and chimpanzees. This lack makes it very difficult to infer inter-chromosomal gene conversions between *VCX *and *VCY *in non-human and non-chimpanzee primates. The most recent gene conversions between *VCX *and *VCY *in humans and chimpanzees are estimated to be 4.2 ± 1.4 and 6.9 ± 1.5 myr ago, respectively (Additional file 4). We identified four *VCX *sequences in the New world monkey genome (data not shown), although no one-to-one orthologous relationship with the four human *VCX *sequences was detected, suggesting species-specific gene duplications or frequent gene conversions within the *VCX *genes on the X chromosome in New world monkey.

## Discussion

### Repetitive sequences in the converted region

Gene conversion is an event in genetic recombination that occurs at high frequencies not only in germ cells through meiotic division but also in somatic cells through mitosis. Recombination occurs between molecules possessing similar nucleotide sequences and is a common repair mechanism for nucleotide lesions in both prokaryotes and eukaryotes. Recombination is initiated by a double-strand break in one gene followed by the repair of this gene by copying the sequence of a similar gene. In the process of this repair, a "Holliday structure" is constructed and depending on the resolution of this structure, the result is called either gene conversion or crossing over [31,32]. However, it is still not clear whether gene conversion between paralogs on the same or different chromosomes proceeds by the same mechanism.

An *L1 *element may play an important role in the repair process. Although the mechanism is not yet well defined, double-strand breaks at chromosomal *LINE *elements can be repaired by gene conversion with another *LINE *element in *cis *or *trans *[33]. Such conversion is often responsible for disease-causing genetic rearrangements [34,35]. In fact, Han et al. [36] identified 73 cases of human-specific deletions associated with recombination between *L1 *elements that have resulted in an approximately 0.5 Mb reduction in the amount of human DNA within the past few million years.

In the present study, we identified a *LINE *element at the boundary of the low *p*-distance region and found that the boundary was located within this *LINE *element (Figure 4). Further, we observed frequent deletions in the primate Y chromosome in the low *p*-distance region. These observations provide evidence of the significant role of repetitive elements, such as a *LINE*, as a source of instability in the region.

At the telomeric end, in contrast, we found a truncated or segmented palindrome, which included three of the four *VCX *members. This palindrome on the X chromosome has a relatively long loop and short arms. Palindrome arms, which contain *VCX *gene family sequences, may have facilitated frequent exchanges with palindrome arms on the Y chromosome [25].

### History of *KAL*s and Kallmann syndrome

A sizeable body of evidence has accumulated to show that, although gene conversion has been an important driving force in human genome evolution, it can also be a cause of inherited diseases [37].

**Figure 6:**
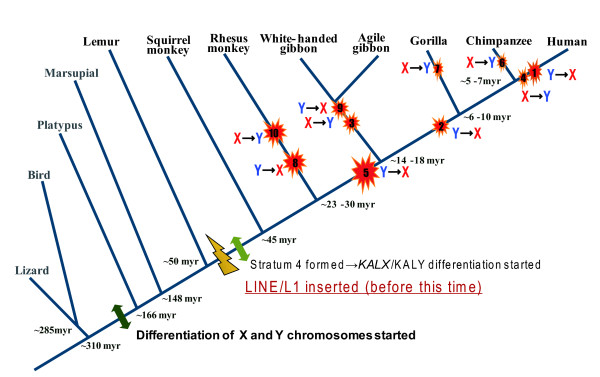
**History of gene conversion in the X and Y homologous chromosomal region**. The hypothesized scenario for *KAL *gene evolution in primates is presented. Before the divergence of Platypus from mammals, the differentiation of sex chromosomes was initiated. After the divergence of prosimian from simian primates, stratum 4 was formed and the differentiation of *KALX *and *KALY *started. There were numerous, independent gene-conversion-like events (at least 10) during the evolution of primates, as indicated by the red stars. The arrows show the direction of conversion. The number in each red star corresponds to the conversion event identified in Figure 3.

Identical *KALX *and *KALY *sequences were located on a pair of ancestral chromosomes before the cessation of recombination in stratum 4 (Figure 6). Both *KALX *and *KALY *were subjected to mutations following the establishment of stratum 4. However, selection pressure removed deleterious mutations from *KALX*, whereas mutations accumulated on *KALY *which subsequently became a pseudogene. Later, frequent gene conversions resulted in partial homogenization between *KALX *and *KALY*, resulting in a reduced number of nucleotide differences between the two genes.

Since *KALY *is a pseudogene and *KALX *is functional, the direction of genetic exchange should be mostly from *KALX *to *KALY*. Gene conversion from *KALY *to *KALX *can potentially destroy the functional *KALX *gene, resulting in Kallmann syndrome. Thus, the conversion from Y to X chromosome after the formation of stratum 4 could lead to Kallmann syndrome. In primate evolution, gene conversion from *KALY *to *KALX *actually occurred as frequently as that from *KALX *to *KALY *(Figure 6). Guioli et al. [38] proposed that exon 14 in *KALX *was replaced homologous segment in *KALY. *This replacement would result in the loss of function in authentic *KALX *and cause the syndrome, suggesting that the translocation of *KALY *onto the X chromosome may be the cause of this replacement. However, the authors did not examine sequences downstream of exon 14 or whether the *KALX *in individuals with Kallmann syndrome is the result of a translocation or the result of gene conversion. The present study raises the possibility that the novel molecular characteristics may indeed affect *KALX *function.

## Conclusions

Genomic sequence analysis revealed a low *p*-distance region in the middle of stratum 4 on the X chromosome of primates. This low *p*-distance was probably caused by ectopic gene conversion in the course of primate evolution and was independently observed in several primate species. Phylogenetic studies of the region revealed at least ten gene conversions within a relatively small ~10 kb region, suggesting the presence of a hot spot for conversions. The examination of primate genome structures reveals the presence of structures that may enhance conversion in the region: large repetitive elements at the centromeric end and a segmented palindrome at the telomeric boundary of this region. This conversion may be one of the causes of Kallmann syndrome.

## Methods

### DNA Samples

Genomic DNA samples were provided from Kyoto University or Max Planck Institute for Biology. These DNA samples were of the following species: chimpanzee (*Pan troglodytes*), gorilla (*Gorilla gorilla*), agile gibbon (*Hylobates agilis*), white-handed gibbon (*Hylobates lar*), rhesus monkey (*Macaca mulatta*), cotton-top tamarin (*Saguinus oedipus*), squirrel monkey (*Saimiri sciureus*), and ring-tailed lemur (*Lemur catta*). The sequenced regions and accession numbers are listed in Additional file 5.

### Gene Amplification and Sequencing

The *KALX *and *KALY *sequences of non-human primates were amplified by PCR using the primers listed in Additional file 6, which were designed based on the human exon sequences of these genes: NM_000216, X68746, X68758, X68759, X68763, X68771, X68762, X68761, and X68770.

PCR was performed in reaction volumes of 20 μl using 20 pmol of each primer, 100 ng of genomic DNA, 10 mM Tris-HCl (pH 8.3), 50 mM KCl, 2 mM MgCl_2_, 200 μM dNTPs, and 2.5 U ExTaq DNA polymerase (TaKaRa). Amplifications were carried out using a RoboCycler Gradient 96 instrument (Stratagene) with the following standard conditions: denaturation at 95°C for 3 min, followed by 30 cycles of 95°C for 30 sec, 56-62°C for 30 sec and 72°C for 6 min, and a final extension at 72°C for 10 min. For rhesus monkeys, we used LA ExTaq DNA polymerase (TaKaRa) to amplify long fragments (>10 kb) with PCR conditions of denaturation at 95°C for 5 min, followed by 30 cycles of 95°C for 1 min, 56-62°C for 1 min and 69°C for 6 min, and a final extension at 69°C for 10 min. These PCR conditions were modified slightly for some primer sets.

All PCR products were purified using a QIAquick PCR Purification Kit (QIAGEN). Some products were directly sequenced while others were cloned into pCR-KL-TOPO using a TOPO XL Cloning kit (Invitrogen). Plasmid DNA was purified with a QIAprep Spin Miniprep column (QIAGEN) and used as the template for sequencing reactions. We used the GeneJumper Primer Insertion Kit for Sequencing (Invitrogen) the long rhesus monkey gene sequences.

Sequencing reactions were performed using an ABI Prism BigDye Terminator Cycle Sequencing FS Ready Reaction Kit (Applied Biosystems) and analyzed on an ABI PRISM 377 DNA sequencer (Applied Biosystems). Sequences for each sample were confirmed by means of independent PCR and subsequent sequencing. To avoid sequencing errors, PCR products or plasmid DNA fragments were sequenced at least twice in both directions. The sequence fragments were assembled by DNASIS (Hitachi) and deposited in the DNA Data Bank of Japan (DDBJ).

### Data Analysis

We obtained 3 kb (exon 5 to 6) and 6 kb (exon 10 to 14) genomic sequences of *KALX *and *KALY *from chimpanzees, gorillas, agile gibbons, white-handed gibbons, squirrel monkeys, and ring-tailed lemurs. For rhesus monkeys, we obtained ~50 kb fragments containing exons 5 to 14 of *KALX *and *KALY*. These rhesus monkey sequences were used for phylogenetic analysis, together with human and chimpanzee genomic orthologous sequences from GenBank. We detected homologous fragments using DOTTER [39], made an alignment using CLUSTALW [40], and adjusted the alignment manually (available upon request). GenBank accession numbers of the sequences retrieved from the database (GenBank) are given in Additional file 5.

We computed *p*-distances (the number of nucleotide differences per site) for all pairwise comparisons of primate sequences. To perform the sliding window analyses of *p*-distances for these sequences, we used our own FORTRAN program (available upon request). We constructed phylogenetic trees using the neighbor-joining (NJ) method [41] and/or the maximum parsimony (MP) method [42] in MEGA4 [43]. The NJ tree was based on *p*-distances in the complete-deletion option and the reliability of the tree was assessed by bootstrap test with 1,000 replications.

### Chromosomal Locations of *KAL*s

The chromosomal locations of *KALX *and *KALY *are known in humans [22]. To map these loci on the chromosomes of chimpanzees, gibbons, rhesus monkeys, squirrel monkeys, and ring-tailed lemurs, we conducted FISH using species-specific *KALX *and *KALY *sequences. Slide preparations of the chromosomes for chimpanzees were made from phytohaemagglutinin-stimulated peripheral white blood cells. Chromosome preparations from gibbons, squirrel monkeys and ring-tailed lemurs were made with fibroblasts [44]. After incubating the slides for a few days at 37°C, FISH was conducted using 200 ng plasmid clones of the *KAL *sequences for each primate as probes. These probes were labeled using a BioNick labeling kit containing biotin-14-dATP (Gibco BRL) [44]. Fluorescent signals were amplified using the sandwich technique [45]. The FISH images were collected using a CCD camera (Photometric) attached to an epifluorescence microscope (Axiophot, Carl Zeiss) and saved on a computer (Macintosh 8500/120) with the IPLab imaging software (Signal Analytics Corporation).

## Abbreviations

(*d*): per-site nucleotide divergence; (*p*): per-site nucleotide sequence differences; (*KALX)*: Kallmann syndrome 1 sequence; *(KALY*): Kallmann syndrome sequence pseudogene; (*VCX*): variable charge, X-linked; (*VCY*): variable charge, Y-linked; (*ZFX)*: zinc finger protein, X-linked; *(ZFY*): zinc finger protein, Y-linked; *(PRKX)*: protein kinase, X-linked; *(PRKY)*: protein kinase, Y-linked

## Authors' contributions

MI designed this study and performed sequencing, phylogenetic analysis and contributed to the writing of this manuscript. YS performed phylogenetic analysis and contributed to the writing of the manuscript. HH and YH performed cytological analyses and NT designed the study. All authors read and approved the final manuscript.

## Supplementary Material

Additional file 1 **Window analysis of nucleotide differences (y-axis) along the X chromosome**. The position of the *KALX *and *VCX *exons are indicated at the bottom of each graph. Each bar corresponds to a *p*-distance in a non-overlapping window of 500 bp. A low *p*-distance region is indicated by the two-headed arrows. The region indicated by the rectangle is enlarged in Figure 1B. Click here for file

Additional file 2 **FISH analysis using species-specific *KALX *and *KALY *probes**. FISH was carried out on metaphase chromosomes from human (*a, b, c *and *d*), chimpanzee (*e, f, g *and *h*), and rhesus monkey (*i, j, k *and *l*). The X (left: *a, b, e, f, i. j*) and Y (Right; *c, d, g, h, k, l*) chromosomes are marked (arrowheads). Green fluorescence indicates a positive signal. A short white bar marks the centromeric region (scale bar: 10 μm). Click here for file

Additional file 3 **The phylogenetic tree of region A and B of *KALX *and *KALY *in several primates**. The trees for Regions A and B are based on *p*-distances. The numbers of sites are given at the bottom right of each tree. A yellow diamond at the node indicates *KALX *and *KALY *differentiation, and the number beside each node is the bootstrap probability after 500 replications. Click here for file

Additional file 4 **The phylogenetic tree of *VCX *and *VCY *in several primates**. Diamonds indicate gene conversion events. Click here for file

Additional file 5 **Sources of DNA samples and GenBank accession numbers of the analyzed sequences**. All the nucleotide sequences reported in this study were deposited in DDBJ (DNA Data Bank of JAPAN) and their accession numbers are AB233497 - AB233526. Click here for file

Additional file 6 **Primer sets for PCR**. Click here for file
