# Supplementary material for: Frequent gene conversion events between the X and Y homologous chromosomal regions in primates
Source: BMC Evol Biol. 2010 Jul 23;10:225. doi: 10.1186/1471-2148-10-225 (PMC3055243; doi:10.1186/1471-2148-10-225)
Supplement: Additional file 5 — Sources of DNA samples and GenBank accession numbers of the analyzed sequences. All the nucleotide sequences reported in this study were deposited in DDBJ (DNA Data Bank of JAPAN) and their accession numbers are AB233497 - AB233526. [file 1471-2148-10-225-S5.DOC]

| species／status | sequence name | accession No. | sequence length(bp) | sequence name | accession No. | sequence length(bp) | Sources |
| --- | --- | --- | --- | --- | --- | --- | --- |
| **chimpanzee (Pan troglodytes) female** | KALX exon5-6 | AB233497 | 2692 | KALX exon10-13 | AB233509 | 7870 | [1] |
| **gorilla (Gorilla gorilla) male** | KALX exon5-6 | AB233498 | 2486 | KALX exon10-14 | AB233510 | 6727 | [1] |
| **gorilla (Gorilla gorilla) female** | KALX exon5-6 |  |  | KALX exon10-14 | AB233512 | 6736 | [1] |
| **agile gibbon (H.agilis) male** | KALX exon5-6 | AB233500 | 2666 | KALX exon10-14 | AB233513 | 6713 | [1] |
| **agile gibbon (H.agilis) female** | KALX exon5-6 | AB233502 | 2662 | KALX exon10-14 | AB233515 | 6610 | [1] |
| **white handed gibbon (H.lar) male2846** | KALX exon5-6 | AB233503 | 2662 | KALX exon10-14 | AB233516 | 6687 | [1] |
| **white handed gibbon (H.lar) male1984** | KALX exon5-6 |  |  | KALX exon10-14 | AB233518 | 6620 | [1] |
| **white handed gibbon (H.lar) female2845** | KALX exon5-6 | AB233505 | 2662 | KALX exon10-14 | AB233520 | 6732 | [1] |
| **white handed gibbon (H.lar) female1982** | KALX exon5-6 |  |  | KALX exon10-14 | AB233521 | 6621 | [1] |
| **cotton-top tamarin (Saguinus oedipus) male** | KALX exon5-6 | AB233506 | 2691 | KALX exon10-14 | AB233522 | 8138 | [1] |
| **squirrel monkey (Saimiri sciureus) male** | KALX exon5-6 | AB233507 | 3036 | KALX exon10-13 | AB233523 | 6099 | [1] |
| **ring-tailed lemur (Lemur catta) male** | KALX exon5-6 | AB233508 | 3011 | KALX exon10-13 | AB233524 | 4736 | [2] |
| **gorilla (Gorilla gorilla) male** | KALY exon5-6 | AB233499 | 2829 | KALY exon10-13 | AB233511 | 5559 | [1] |
| **agile gibbon (H.agilis) male** | KALY exon5-6 | AB233501 | 2632 | KALY exon10-14 | AB233514 | 6983 | [1] |
| **white handed gibbon (H.lar) male2846** | KALY exon5-6 | AB233504 | 2714 | KALY exon10-14 | AB233517 | 7003 | [1] |
| **white handed gibbon (H.lar) male1984** | KALY exon5-6 |  |  | KALY exon10-14 | AB233519 | 6937 | [1] |
| **rhesus monkey (Macaca mulatta) male** | KALX  exon5-14 | AB233525 | 57463 |  |  |  | [1] |
| **rhesus monkey (Macaca mulatta) male** | KALY  exon5-14 | AB233526 | 49298 |  |  |  | [1] |

[1]Primate Research Institute, Kyoto University, Inuyama, Japan.

[2]Department of Immunogenetics, Max Planck Institute for Biology,　Tübingen,　Germany
