# Supplementary material for: Frequent gene conversion events between the X and Y homologous chromosomal regions in primates
Source: BMC Evol Biol. 2010 Jul 23;10:225. doi: 10.1186/1471-2148-10-225 (PMC3055243; doi:10.1186/1471-2148-10-225)
Supplement: Additional file 6 — Primer sets for PCR. [file 1471-2148-10-225-S6.DOC]

| **species／status** | **Exon 5 (forward) Exon 6 (reverse)** | | **Exon 10 (forward) Exon 14 (reverse)** |
| --- | --- | --- | --- |
| **chimpanzee (Pan troglodytes) female** | (forward)GGTGTCCCCCTGAAGCCCA (reverse)ACGGAAGTGTTTGCTGGGGG | | (forward)AATTTCATCAAATAGTTTCTCTT (reverse)AACCAAGGGCTGGGGCAAGGGCA |
| **gorillas (Gorilla gorilla)**  **male** | (forward)CACTCGAAACTGGTACCATC (reverse)ACTCATTGGCAGACGGTGG | | (forward)TCCCACTGTCAACCGATATCATG (reverse)TGGAGAAGGCTTGTAATGATGTGG |
| **gorilla (Gorilla gorilla)**  **female** |  | | (forward)TCCCACTGTCAACCGATATCATG (reverse)TGGAGAAGGCTTGTAATGATGTGG |
| **agile gibbon (H.agilis)**  **male** | (forward)GGTGTCCCCCTGAAGCCCA (reverse)ACGGAAGTGTTTGCTGGGGG | | (forward)TCCCACTGTCAACCGATATCATG (reverse)TGGAGAAGGCTTGTAATGATGTGG |
| **agile gibbon (H.agilis)**  **female** | (forward)GGTGTCCCCCTGAAGCCCA (reverse)ACGGAAGTGTTTGCTGGGGG | | (forward)GAGACATCATCTGGCACGACTCA (reverse)ATGAGATCCTAAAAAGTGACAAAATA |
| **white handed gibbons (H.lar) male2846** | (forward)GGTGTCCCCCTGAAGCCCA (reverse)ACGGAAGTGTTTGCTGGGGG | | (forward)GTGCCCACAACAGAACAACCGGA (reverse)TGGAGAAGGCTTGTAATGATGTGGATGA |
| **white handed gibbon (H.lar) male1984** |  | | (forward)GAGACATCATCTGGCACGAC (reverse)ATGAGATCCTAAAAAGTGACAAA |
| **white handed gibbon (H.lar) female2845** | (forward)GGTGTCCCCCTGAAGCCCA (reverse)ACGGAAGTGTTTGCTGGGGG | | (forward)TCCCACTGTCAACCGATATCATGT (reverse)TGGAGAAGGCTTGTAATGATGTGGATGA |
| **white handed gibbons (H.lar) female1982** |  | | (forward)GAGGCATCATCTGGCACGAC (reverse)ATGAGATCCTAAAAAGTGACAAA |
| **cotton-top tamarin**  **(Saguinus oedipus) male** | (forward)GGTGTCCCCCTGAAGCCCA (reverse)ACGGAAGTGTTTGCTGGGGG | | (forward)TCCCACTGTCAACCGATATCATG (reverse)TGGAGAAGGCTTGTAATGATGTGG |
| **squirrel monkey**  **(Saimiri sciureus) male** | (forward)GGTGTCCCCCTGAAGCCCA (reverse)ACGGAAGTGTTTGCTGGGGG | | (forward)GTGCCCACAACAGAACAACCGG (reverse)TTCCCTCGCTCTCATTAGACACTG |
| **ring-tailed lemur**  **(Lemur catta) male** | (forward)GGTGTCCCCCTGAAGCCCA (reverse)ACGGAAGTGTTTGCTGGGGG | | (forward)GTGCCCACAACAGAACAACCGGA (reverse)GATGGTCTCCGATTGGGCACTGTT |
| **gorilla (Gorilla gorilla)**  **male** | (forward)ACTCATTGGCAGACGGTGG (reverse)TGATGGTACCAGTTTCGAGTG | | forward)GTGCCCACAACAGAACAACCGGA (reverse)GATGGTCTCCGATTGGGCACTGTT |
| **agile gibbon (H.agilis)**  **male** | (forward)GAAGGGTTACAATTTACAGAA (reverse)TGGAATGTGCATGGAACTCG | | (forward)GAGGCATCATCTGGCACGACTCATG (reverse)TGGAGAAGGCTTGTAATGATGTG |
| **white handed gibbon (H.lar) male2846** | (forward)GGTGTCCCCCTGAAGCCCA (reverse)ACGGAAGTGTTTGCTGGGGG | | (forward)GTGCCCACAACAGAACAACCGGA (reverse)GAAGGCTTGTAATGATGTGGATGA |
| **white handed gibbon (H.lar) male1984** |  | | (forward)GAGGCATCATCTGGCACGACTC (reverse)ATGAGATCCTAAAAAGTGACAAA |
| **rhesus monkey (Macaca mulatta) male KALX exon5-14** | | **rhesus monkey (Macaca mulatta) male KALY exon5-14** | |
| Exon 5 (forward)GGTGTCCCCCTGAAGCCCA Exon 6 (reverse)ACGGAAGTGTTTGCTGGGGG | | Exon 5(forward)GGTGTCCCCCTGAAGCCCA Exon 6(reverse)ACGGAAGTGTTTGCTGGGGG | |
| Intron 5 (forward)GGCTGATAGGTTAGTATATTTTTC Exon 7(reverse)CAGGAAATAGCGTGGGAACC | | Intron 5(forward)TTCATAACTGGACGTGATAAGCAGCG Exon 7(reverse)GGAGTTGGCCAATCGGAGGTTAGC | |
| Intorn 6(forward)GAGCCACCATGCCCCTTCACCCCCGT Exon 8(reverse)ATGTGTGGATGTGAAGTGAAGGGAC | | Exon 7 (forward)GCTAACCTCCGGCTGGCCAACTCC Exon9 (reverse)AGACTTTAACTTGCATTTGGCCATCCTGAT | |
| Exon 8(forward)ACGGCTGAAGAGTGCGAAGGTGTCCC Exon 9(reverse)GACTTT AACTTGCAT TTGGCCATCCTGATA | | Exon 9 (forward)AACAAGACAAAATGAAGTACACTGGGAC Exon 13 (reverse)CAGCACTTGCACTACAGACGGT | |
| Exon9 (forward)CTATCAGGATGGCCAAATGCAAG Exon 10 (reverse)ACACTTCAGGAAACCACCGCACA | | Exon 9 (forward)ATGACCTTAGAATAGCCAAATGCCAC  Exon 13 (reverse)AGGGTGGCTGTAGAAATGAATAAAGGAA | |
| Exon10 (forward)TCCCACTGTCAACCGATATCATGT Exon 14 (reverse)TGGAGAAGGCTTGTAATGATGTGGATGA | |  | |
| Exon 13 (forward)AGCCAACTCATGAGAAGACCTTACCC  flanking (reverse)AAGTGGGGATGTACAAGCCTCTCAAA | |  | |
